# Gghic: a versatile R package for exploring and visualizing 3D genome organization

**DOI:** 10.1093/bib/bbaf631.042

**Published:** 2025-12-12

**Authors:** Minghao Jiang, Duohui Jing, Jason WH Wong

**Affiliations:** School of Biomedical Sciences, Li Ka Shing Faculty of Medicine, The University of Hong Kong, Hong Kong SAR, China; Shanghai Institute of Hematology, State Key Laboratory of Medical Genomics, Ruijin Hospital Affiliated to Shanghai Jiao Tong University School of Medicine, Shanghai, China; School of Biomedical Sciences, Li Ka Shing Faculty of Medicine, The University of Hong Kong, Hong Kong SAR, China

## Abstract

Three-dimensional genome architecture is essential for gene regulation and cellular homeostasis. Its perturbation underlies pathologies. Advances in chromosome conformation capture technologies, such as Hi-C [1], have enabled researchers to study genome architecture at high resolution, yet the resulting matrices are difficult to explore visually, especially when one wishes to overlay genomic annotations or examine inter-chromosomal interactions.

We introduce gghic, an R package that extends the ggplot2 grammar for the publication-quality visualization of chromatin-interaction data. Gghic supplies layers that draw (i) triangular Hi-C heatmaps, (ii) chromatin loops, topologically associating domains, (iii) gene or transcript models, (iv) one-dimensional signal tracks, (v) and multi-way contacts. The package supports Bioconductor classes, including HiCExperiment [2] and GInteractions [3]. It handles data from multiple chromosomes, enabling inspection of inter-chromosomal contacts.

We illustrate the utility of gghic with acute lymphoblastic leukemia Hi-C datasets, producing publication-ready figures that reveal translocation breakpoints and higher-order chromatin hubs. By coupling advanced genome-structure analyses with the ggplot2 syntax, gghic makes the exploration and communication of 3D genomics data both intuitive and reproducible.

**References:**

1. Belton JM, McCord RP, Gibcus JH et al. ‘Hi-C: a comprehensive technique to capture the conformation of genomes’ Methods 2012;58:268–276.

2. Serizay J, Matthey-Doret C, Bignaud A et al. ‘Orchestrating chromosome conformation capture analysis with Bioconductor’ Nat Commun 2024;15:1072.

3. Lun AT, Perry M, Ing-Simmons E. ‘Infrastructure for genomic interactions: Bioconductor classes for Hi-C, ChIA-PET and related experiments’ F1000Res 2016;5:950.

